# Altered Causal Connectivity of Resting State Brain Networks in Amnesic MCI

**DOI:** 10.1371/journal.pone.0088476

**Published:** 2014-03-10

**Authors:** Peipeng Liang, Zhihao Li, Gopikrishna Deshpande, Zhiqun Wang, Xiaoping Hu, Kuncheng Li

**Affiliations:** 1 Department of Radiology, Xuanwu Hospital, Capital Medical University, Beijing, China; 2 Beijing Key Laboratory of Magnetic Resonance Imaging and Brain Informatics, Beijing, China; 3 Key Laboratory for Neurodegenerative Diseases, Ministry of Education, Beijing, PR China; 4 Wallace H Coulter Department of Biomedical Engineering, Georgia Institute of Technology and Emory University, Atlanta, Georgia, United States of America; 5 Auburn University MRI Research Center, Department of Electrical and Computer Engineering, and Department of Psychology, Auburn University, Auburn, Alabama, United States of America; Beijing Normal University, China

## Abstract

Most neuroimaging studies of resting state networks in amnesic mild cognitive impairment (aMCI) have concentrated on functional connectivity (FC) based on instantaneous correlation in a single network. The purpose of the current study was to investigate effective connectivity in aMCI patients based on Granger causality of four important networks at resting state derived from functional magnetic resonance imaging data – default mode network (DMN), hippocampal cortical memory network (HCMN), dorsal attention network (DAN) and fronto-parietal control network (FPCN). Structural and functional MRI data were collected from 16 aMCI patients and 16 age, gender-matched healthy controls. Correlation-purged Granger causality analysis was used, taking gray matter atrophy as covariates, to compare the group difference between aMCI patients and healthy controls. We found that the causal connectivity between networks in aMCI patients was significantly altered with both increases and decreases in the aMCI group as compared to healthy controls. Some alterations were significantly correlated with the disease severity as measured by mini-mental state examination (MMSE), and California verbal learning test (CVLT) scores. When the whole-brain signal averaged over the entire brain was used as a nuisance co-variate, the within-group maps were significantly altered while the between-group difference maps did not. These results suggest that the alterations in causal influences may be one of the possible underlying substrates of cognitive impairments in aMCI. The present study extends and complements previous FC studies and demonstrates the coexistence of causal disconnection and compensation in aMCI patients, and thus might provide insights into biological mechanism of the disease.

## Introduction

Alzheimer's disease (AD) is the most prevalent form of dementia worldwide with symptoms of global cognitive decline, including progressive loss of memory, reasoning and language. The neuropathological changes of AD are characterized by amyloid-β plaques, neurofibrillary tangles and neuronal loss [1]. Amnesic mild cognitive impairment (aMCI) is an intermediate state between healthy aging and AD, with a higher risk of developing dementia (rate of conversion of 10–15% per year) [2]. There has been much anatomical and functional neuroimaging evidence characterizing AD as a neural disconnection syndrome [3]–[8]. This connectivity impairment suggests the existence of abnormal interactions within and between neuronal systems in AD [5]. Therefore, it is of significance to evaluate whether the connectivity profiles are affected at the aMCI stage. If so, it could potentially lead to an early diagnosis marker of AD.

Resting state functional magnetic resonance imaging (rs-fMRI) is especially applicable to the study of patients because of the practical advantages it offers in terms of the patients not being required to perform any task. Recently, many rs-fMRI studies have been conducted to investigate the pathogenesis of MCI and AD. They are all primarily based on characterizing functional connectivity within a given network, such as the default mode network (DMN) [9]–[16], hippocampal cortical memory network (HCMN) [7], [17]–[19], task-positive network (TPN) [20], executive control network (ECN) and salience network (SN) [21]. Using seed-based functional connectivity (FC) and independent component analysis (ICA), these studies demonstrated the abnormalities of functional integrity in MCI patients [9], [11], [16] and showed that functional disconnection and compensation coexisted in MCI patients. However, two shortcomings stand out in previous studies. First, most of the previous work investigated only the connectivity in one single network and did not investigate connectivity between multiple networks. Second, most of the previous work investigated only FC in these networks, which does not provide information regarding the direction of connectivity. Previous studies have shown that incorporating resting state effective connectivity (EC), in additional to functional connectivity, increases diagnostic classification accuracy [22]. Therefore investigating directional interactions within and between these networks using data driven EC techniques such as granger causal analysis (GCA) may provide new insights into the underlying network alterations in aMCI.

There have been several studies focused on the effective connectivity of brain networks in AD, using multivariate Granger causality analysis (mGCA) [23]–[24] or the sparse Bayesian Network (BN) [25]–[26]. These studies identified both decreased and increased EC in AD versus healthy controls, which was ascribed to the dysfunctional and compensatory processes in AD. However, two studies investigated effective connectivity only among regions of the DMN [24]–[25]. Although the other two studies report EC in different resting state networks, they had one time series derived from independent component analysis (ICA) representing the entire network, thereby loosing spatial specificity [23], [26]. In particular, the sparse literature on EC analysis of resting state networks [27]–[28] ignored the “leakage” of instantaneous correlation into estimates of causality [29].

To the best of our knowledge, no study has been done on the EC of brain networks in MCI/aMCI patients. In this work, we address the limitations in previous studies of resting state brain networks in MCI patients. First, we examined the connectivity patterns within and between four important brain networks, including the DMN [30]–[31], the dorsal attention network (DAN) [32], the fronto-parietal control network (FPCN) [33] and the HCMN [33]. These functional brain networks have been confirmed by using resting state functional connectivity, as measured by Pearson's correlation in low frequency fluctuations (LFF) (<0.1 Hz) of the blood oxygen level-dependent (BOLD) signal. Of particular interest are DMN and DAN, since the former is engaged by internally directed cognition and the latter by externally directed cognition, and hence are anti-correlated [34]–[35]. It has been hypothesized that FPCN mediates the interaction between DMN and DAN [33], [36], given the evidence that it is anatomically juxtaposed between DMN and DAN. As aMCI patients have evident memory impairment, it is of direct interest to examine the connectivity with HCMN and the interactions between HCMN and the other three basic networks. We will define the functional regions of interest (ROI) of four networks based on coordinates in previous published literatures to obtain time series specific to each ROI, unlike previous ICA-based studies which obtained a single time series for the entire network. This allowed us to examine the intra- and inter- networks connectivity without losing spatial specificity.

Second, using the analysis of correlation-purged Granger causality (CPGC) [29], [37]–[38], we were able to ascertain the causal connectivity between all the ROIs in the four networks. Due to the blurring of the neuronal activity with the sluggish hemodynamic response, interaction between the instantaneous and time-lagged relationships between time series can be observed in experimental fMRI data. The “leakage” of the zero-lag effects into time-lagged estimates can result in confounding of the causality obtained from the VAR model, by instantaneous correlation between time series. In order to alleviate this potential confound, CPGC method was introduced [29]. In comparison with confirmatory approaches such as dynamic causal modeling (DCM) and structural equation modeling (SEM), which require a priori specification of the underlying connectivity architecture, CPGC is an exploratory technique. Therefore, we were able to obtain directional connectivity between the ROIs without making any *a priori* assumptions about the connectivity between them.

Many neuroimaging studies have shown that cognitive and memory decline in patients with aMCI is coupled with abnormal functions of focal brain regions [39]–[43], aberrant functional connectivity between distinct brain regions [10]–[12], [16], [19], [20]–[21], as well as a disruption of whole-brain topological organization of the functional connectome [44]. Based on these previous findings on aMCI, and also referring to AD-related studies [7], [15], [24]–[25], we hypothesized that the directional connectivity profiles between resting state brain networks in aMCI patients may be altered in two opposite directions, i.e., the causal disconnection and compensation may coexist in aMCI patients as compared to healthy controls.

## Materials and Methods

### Subjects

This study was approved by the Medical Research Ethics Committee of Xuanwu Hospital. Thirty-six right-handed subjects (18 aMCI patients and 18 healthy elders) participated in this study, and written informed consent was obtained from each participant. The aMCI subjects were recruited from patients who had consulted a memory clinic for memory problems at Xuanwu Hospital, Beijing, China. The healthy elderly controls were recruited from the local community through advertisements.

Participants with aMCI had memory impairment but did not meet the criteria for dementia. The criteria for identification and classification of subjects with MCI [45] was: (a) impaired memory performance on a normalized objective verbal memory test; (b) recent history of symptomatic worsening in memory; (c) normal or near-normal performance on global cognitive tests [Mini-Mental State Examination (MMSE) score >24], as well as on activities of daily living scale; (d) global rating of 0.5 on the clinical dementia rate (CDR) Scale, with a score of at least 0.5 on the memory domain; (e) absence of dementia.

The criteria for healthy elderly controls were as follows: (a′) matched with aMCI patients by the gender, age and education level; (b′) no neurological or psychiatric disorders such as stroke, depression, epilepsy; (c′) no neurological deficiencies such as visual or hearing loss; (d′) no abnormal findings such as infarction or focal lesion in conventional brain MR imaging; (e′) no cognitive complaints; (f′) MMSE score of 28 or higher; (g′) CDR score of 0.

The exclusion criteria were as follows: all subjects with contra-indications to MRI such as pacemaker, cardiac defibrillator, implanted material with electric or magnetic system, vascular clips or mechanical heart valve, cochlear implant or claustrophobia were excluded; stroke, psychiatric diseases, drug abuse, moderate to serious hypertension, and systematic diseases were ruled out; persons with intellectual disability were also not included in the group.

Data from four subjects (2 aMCI patients and 2 healthy elders) were excluded due to excessive motion (see Image preprocessing). Clinical and demographic data for the remaining 32 participants are shown in Table 1.

**Table 1:** Demographics and clinical findings.

	aMCI(*N* = 16)	Controls(*N* = 16)	*p* value
Sex, female/male	10/6	10/6	>0.99#
Age, year	68.50±7.77	67.19±8.38	0.506*
Education, year	10.06±3.91	9.06±3.64	0.857*
MMSE^a^	25.94±1.65	28.56±0.63	<0.05*
CVLT(immediate)	8.39±1.23	11.33±1.86	<0.05*
CVLT(short time)	9.13±1.23	13.00±1.86	<0.05*
CVLT(long-time)	7.31±1.43	12.62±1.36	<0.05*
CDT	5.75±0.54	8.62±1.36	<0.05*

MMSE, Mini-Mental State Examination; values are means ± SD. WHO-UCLA CVLT, California verbal learning test; immediate, immediate recall of learning verbal; short-time, short time delayed free recall; long-time, long time delayed free recall; CDT, clock drawing test.

# The p value was obtained using a Pearson x^2^ two-tailed test, with continuity correction for n<5.

* The p value was obtained by a two-sample two-tailed t-test.

### MRI Data Acquisition

MRI data were acquired on a SIEMENS Trio 3-Tesla scanner (Siemens, Erlangen, Germany). Foam padding and headphones were used to limit head motion and reduce scanner noise. The subjects were instructed to hold still, keep their eyes closed and think nothing in particular. Functional images were collected axially by using an echo-planar imaging (EPI) sequence [repetition time (TR)/echo time (TE)/flip angle (FA)/field of view (FOV) = 2000 ms/40 ms/90°/24 cm, resolution = 64×64 matrix, slices = 28, thickness = 4 mm, gap = 1 mm, bandwidth = 2232 Hz/pixel]. The scan lasted for 478 s. 3D T1-weighted magnetization-prepared rapid gradient echo (MPRAGE) sagittal images were collected by using the following parameters: TR/TE/inversion time (TI)/FA = 1900 ms/2.2 ms/900 ms/9°, resolution = 256×256 matrix, slices = 176, thickness = 1.0 mm.

### FMRI Data Preprocessing

Unless otherwise stated, all analyses were conducted using statistical parametric mapping software package (SPM5, http://www.fil.ion.ucl.ac.uk/spm). The first 10 volumes of the functional images were discarded to aloow the signal to reach equilibrium and participants' adaptation to the scanning noise. The remaining 229 fMRI images were first corrected for within-scan acquisition time differences between slices and then realigned to the first volume to correct for inter-scan head motion. No participant had head motion of more than 1.5 mm maximum displacement in any of the x, y, or z directions and 1.5° of any angular motion throughout the course of scan. The individual structural image was co-registered to the mean functional image after motion correction using a linear transformation. The transformed structural images were then segmented into gray matter (GM), white matter (WM) and cerebrospinal fluid (CSF) by using a unified segmentation algorithm [46]. The motion corrected functional volumes were spatially normalized to the Montreal Neurological Institute (MNI) space and re-sampled to 3 mm isotropic voxels using the normalization parameters estimated during unified segmentation. Subsequently, the functional images were spatially smoothed with a Gaussian kernel of 4×4×4 mm^3^ full width at half maximum (FWHM) to decrease spatial noise. Following this, temporal filtering (0.01 Hz<f<0.08 Hz) was applied to the time series of each voxel to reduce the effect of low-frequency drifts and high-frequency noise [30], [47] using Resting-State fMRI Data Analysis Toolkit (http://resting-fmri.sourceforge.net). To further reduce the effects of confounding factors, we also regressed out the following sources of confounds [34]: (1) six motion parameters, (2) linear drift, (3) the white matter signal, (4) the cerebral spinal fluid (CSF) signal.

### ROI Definition

**Figure 1:**
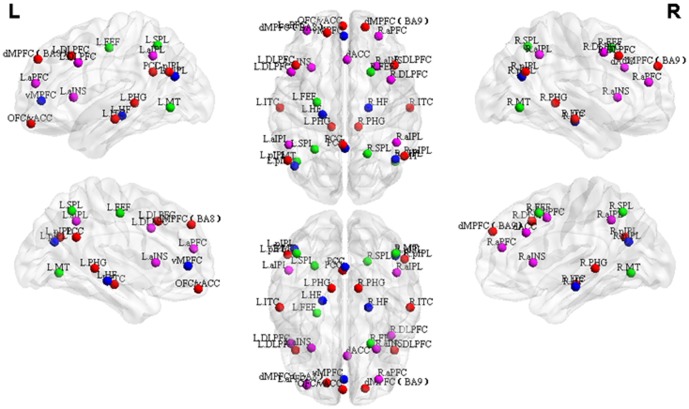
The spatial distributions of 33 ROIs from DMN, HCMN, DAN and FPCN. The coordinates of ROIs are shown in Table 2. The abbreviations are as described in the main text.

Table 2 lists the Talairach coordinates of the ROIs of all 4 networks and Figure 1 shows the corresponding spatial distributions. Coordinates of 33 ROIs from 4 resting state brain networks – DMN, HCMN, DAN, FPCN –were defined according to peer-reviewed published literatures. The ROIs of DMN were defined according to Greicius et al. [30], which included posterior cingulate cortex (PCC), left/right posterior inferior parietal lobule (L/R pIPL), orbitofrontal cortex/ventral anterior cingulate cortex (OFC/vACC), dorsomedial prefrontal cortex Brodmann area 8 (dMPFC BA8), dorsomedial prefrontal cortex Brodmann area 9 (dMPFC BA9), left dorsolateral prefrontal cortex (L DLPFC), left parahippocampal gyrus (L PHG) and left inferolateral temporal cortex (L ITC). The ROIs of HCMN, DAN and FPCN were chosen based on coordinates reported by Vincent et al [33]. Using a sub-function of the “GingerALE” software package (http://brainmap.org/ale/) called “icbm2tal”, the coordinates in Vincent et al [33] were transformed from MNI space to Talairach space. Accordingly, DAN was composed of left/right middle temporal area (L/R MT), left/right frontal eye fields (L/R FEF) and left/right superior parietal lobule (L/R SPL), HCMN consisted of left/right hippocampal formation (L/R HF), ventromedial prefrontal cortex (vmPFC), PCC, and bilateral pIPL, and FPCN consisted of bilateral anterior prefrontal cortex (aPFC), dorsal anterior cingulate cortex (dACC), left/right dorsolateral prefrontal cortex (L/R DLPFC), left/right anterior insula (L/R aINS) and left/right anterior inferior parietal lobule (L/R aIPL). In order to distinguish the regions common to DMN and HCMN we have used the corresponding network names as their suffix (for example, PCC DMN and PCC HCMN). ROIs were 12 mm spheres centered at the corresponding coordinates and were masked by a template consisting of only those voxels which were inside the brain using WFU_PickAtlas toolbox (www.ansir.wfubmc.edu). Consequently, the sizes of some of the ROIs differed slightly.

**Table 2:** The Talairach coordinates of the selected ROIs in DMN, HCMN, DAN and FPCN. The abbreviations are as described in the main text.

Networks	Peak Talairach Coordinates		
	x	y	z
*Default Mode Network*			
PCC	−2	−51	27
L pIPL	−51	−65	27
R pIPL	53	−61	27
OFC/vACC	−2	55	−18
dMPFC BA 8	−16	49	38
dMPFC BA 9	18	54	32
L DLPFC	−44	20	41
R DLPFC	44	20	41
L PHG	−12	−35	0
R PHG	12	−35	0
L ITC	−58	−18	−14
R ITC	58	−18	−14
*Hippocampal Cortical Memory Network*			
L HF	−20	−24	−11
R HF	21	−18	−16
vMPFC	−1	46	2
PCC	0	−54	14
L pIPL	−45	−70	23
R pIPL	45	−64	23
*Dorsal Attention Network*			
L MT	−43	−66	−4
R MT	45	−66	−4
L FEF	−25	−13	48
R FEF	23	14	48
L SPL	−27	−55	50
R SPL	20	−59	49
*Fronto-parietal Control Network*			
L aPFC	−34	51	17
R aPFC	30	46	18
dACC	2	25	31
L DLPFC	−48	14	35
R DLPFC	41	7	44
L aINS	−30	18	5
R aINS	28	19	5
L aIPL	−50	−51	41
R aIPL	46	−49	42

### Regional GM Atrophy

Brain atrophy may cause a partial volume effect in functional imaging techniques [48]. Recent researches have demonstrated the potential impact of atrophy on the functional results in previous fMRI studies of AD [49]–[50] and MCI [10], [20]. To control for the potential impact of atrophy on the functional results, a voxel-based morphometry analysis of structural images was performed. GM intensity maps were obtained by the unified segmentation algorithm [46] as described in the Data preprocessing section. After spatially smoothing with a Gaussian kernel of 10 mm FWHM, a two-sample t-test was performed on the smoothed GM intensity maps to examine regional GM atrophy in MCI patients as compared to healthy controls. The statistical threshold was set at p<0.001 and cluster size >324 mm^3^, which corresponded to a corrected p<0.05 (using the AlphaSim program with parameters: FWHM = 10 mm, within the GM mask).

### Directional Connectivity Analysis

Suppose *x_n_*, *n* = *1 … k* corresponds to the *k* selected ROI time series and ***X*** *(t)* = (*x_1_(t),x_2_(t)*… *x_k_(t)*)^T^, then the modified multivariate autoregressive (mMVAR) model used by Deshpande et al for calculating correlation-purged Granger causality (CPGC) [37] is as follows:
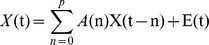
where ***A*** *(n)* are model coefficients of order *p*, and ***E*** *(t)* is the vector corresponding to the residual error. In order to model only the instantaneous cross-correlation between time series, but not the auto-correlation of individual time series, the diagonal elements of *A(0)* were set to zero. The causal relationship between ROI time series is contained in model coefficients *A (1)* … *A (p)*. Additionally, since instantaneous cross-correlation is modeled out in *A(0)*, *A (1)* … *A (p)* are purged of correlation leakage effects and is termed as CPGC. On the other hand, the off-diagonal elements of *A(0)* correspond to zero-lag correlation. Direct causal relationship between the *k* selected ROI time series can be inferred from the model parameters as follows:
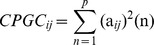
where *a_ij_* are the elements of the matrix ***A*** and *CPGC_ij_* corresponds to the direct causal influence exerted from ROI *j* to ROI *i*.

All the steps of CPGC analysis we followed here are identical to what is reported in Deshpande et al. [51]. First, the time series corresponding to all 33 ROIs of the four networks were extracted, standardized for each ROI and subject, and then input into a fifth order mVAR model [29], [37]–[38] to obtain the instantaneous and causal connectivity between them. The order of the mVAR model was determined using Bayesian Information Criterion (BIC) [52]. Surrogate data were obtained from the ROI time series by randomizing their phase, but retaining the magnitude spectrum, and input into the mVAR model. The above procedure was repeated 10,000 times to derive empirical null distributions for each path.

### Statistics

The within-group statistical significance of the connectivity paths was obtained using fisher's method by comparing the CPGC value obtained from the original data with the empirical null distributions [53]–[54]. If any pair of ROIs significantly influenced each other (p<0.01) in both directions, the two ROIs were considered bi-directionally connected. On the other hand, if only one of the ROIs in a pair had a significant causal interaction on the other but not vice versa, then the former was deemed to have a unidirectional causal influence on the latter.

The CPGC values were then entered into a one-way ANOVA test to identify the significant between-group differences. To control for the potential impact of atrophy on the functional results, the VBM results were included as a covariate. The causal paths (bi-directional or uni-directional) surviving a threshold of P<0.01 (with the corresponding within-group significant causal paths as masks), were considered to show significant difference between aMCI patients and healthy controls. Thus, the significantly increased causal paths in aMCI, as compared to NC, were the intersection of the paths with significant aMCI>NC and the significant within-group paths of aMCI. The significantly decreased paths in aMCI, as compared to NC, were the intersection of the paths with significant aMCI<NC and the significant within-group paths of NC.

### Connectivity-behavior Correlation

In order to explore whether the alterations of causal connectivity covaried with disease progression, a correlation analysis between the CPGC values and neuropsychological performance metrics was performed for aMCI patients. First, CPGC values of the paths with the significant group differences were extracted. Then, Pearson's correlative analysis were performed to examine relationships between CPGC values and neuropsychological performances (including CVLT: Immediate Recall, CVLT: Short Delayed Recall, CVLT: Long Delayed Recall, CDT, and MMSE) in MCI patients using SPSS software (SPSS, Inc., Chicago, IL).

## Results

There were no significant differences between aMCI patients and healthy controls in gender, age, and years of education, but the MMSE, CVLT and CDT scores were significantly different (*P*<0.05) between the two groups. The head motion during the MRI scanning was also not significantly between the two groups (*P*>0.05).

As compared to healthy controls, aMCI patients showed many regions with significant gray matter loss in the right inferior frontal gyrus (IFG), bilateral superior frontal gyrus, right inferior temporal gyrus, right insula, bilateral inferior parietal lobule (IPL), superior parietal lobule, precuneus, middle occipital gyrus, and fusiform gyrus. The global maximum of atrophy was located in the precuneus. These results are consistent with previous studies (see two meta-analyses [55]–[56]).

### Causal Connectivity: Between-group Differences

**Figure 2:**
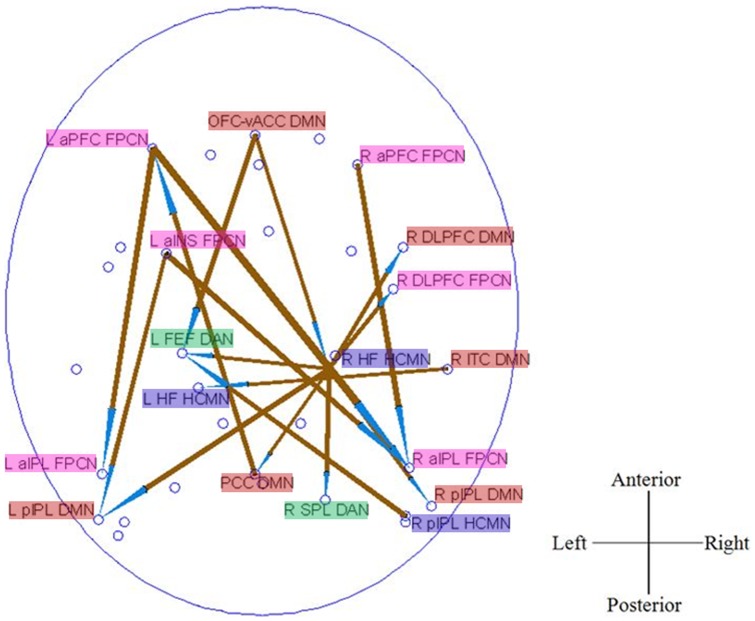
Between group differences of causal connectivity: NC>aMCI. The arrow of the significant causal paths represents the direction of the information flow, and the thickness represents the strength of the causal connectivity.

Figure 2 shows the significantly decreased causal connections in aMCI patients as compared to healthy controls. Three main effects can be observed in this pattern. First, there are evident anterior-to-posterior disconnections within FPCN, such as L aPFC→L aIPL, L aPFC→R aIPL, and R aPFC→R aIPL. Second, the pathways, specifically the output pathways, of right hippocampus (HCMN) were disrupted, including R HF→L FEF, L pIPL, PCC, R SPL, R pIPL (DMN), R aIPL (FPCN), R DLPFC (FPCN), R DLPFC (DMN), together with a decreased input pathway of OFC-vACC→R HF. Third, the causal connections between different networks were impaired, such as OFC-Vacc (DMN)→L FEF (DAN), L aINS (FPCN)→L pIPL (DMN), PCC (DMN)→L aPFC (FPCN), R ITC (DMN)→L HF (HCMN), R HF (HCMN)→PCC (DMN), R pIPL (HCMN)→L FEF (DAN), and R HF (HCMN)→R DLPFC (FPCN).

**Figure 3:**
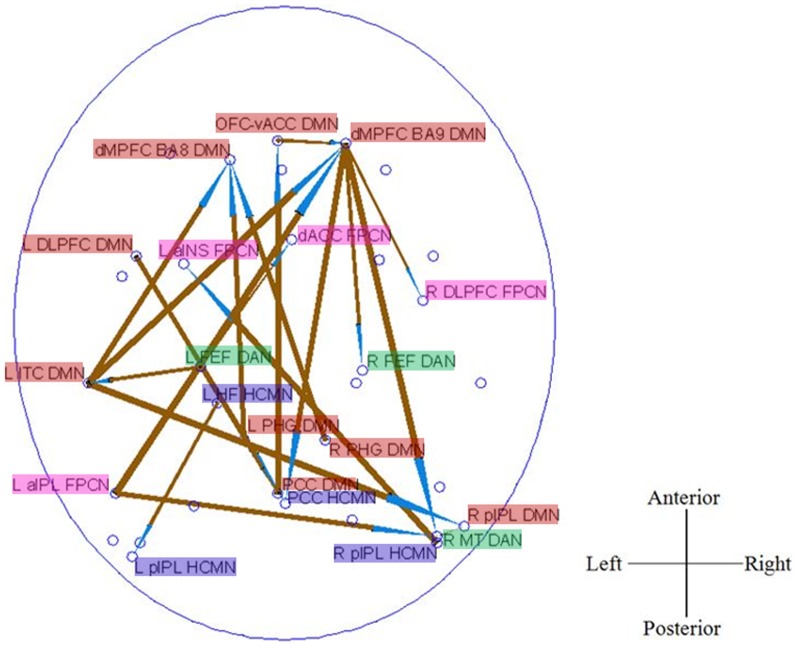
Between group differences of causal connectivity: aMCI>NC. The arrow of the significant causal paths represents the direction of the information flow, and the thickness represents the strength of the causal connectivity.

Figure 3 shows the significantly increased causal connections in aMCI patients as compared to healthy controls. Three primary effects can be observed from Fig. 2. First, the long-distance (anterior-posterior) causal links in which the medial DMN nodes (including dMPFC BA9 DMN, dMPFC BA8 DMN, and PCC DMN) serve as “hubs”, such as L PHG DMN→dMPFC BA8 DMN. R PHG DMN→dMPFC BA8 DMN, PCC DMN→OFC-vACC DMN, dMPFC BA9 DMN→PCC HCMN, dMPFC BA9 DMN→R pIPL HCMN, and L aIPL FPCN→dMPFC BA9 DMN, were higher in aMCI patients. Moreover, increased causal connectivity between a lateral node of DMN located at L ITC (DMN) and other areas, such as L ITC→dMPFC BA8 DMN, dMPFC BA9 DMN, R pIPL DMN, was found. These results point to a significantly increased causal connectivity pattern in aMCI group within the DMN. Second, we observed the short-distance causal connections within the right frontal cortex, including OFC-vACC DMN→dMPFC BA9 DMN, dMPFC BA9 DMN→R DLPFC FPCN, and dMPFC BA9 DMN→R FEF DAN higher in aMCI. Third, we found many between-networks causal connections, including L aIPL FPCN→R MT DAN, L FEF DAN→dACC FPCN, R pIPL HCMN→L aINS FPCN, dMPFC BA9 DMN→R FEF DAN, L aIPL FPCN→dMPFC BA9 DMN, etc. Additionally, we also observed an increased connectivity within HCMN, i.e., L HF HCMN→L pIPL HCMN.

### Correlations between Causal Connectivity and Neuropsychological Measures

**Figure 4:**
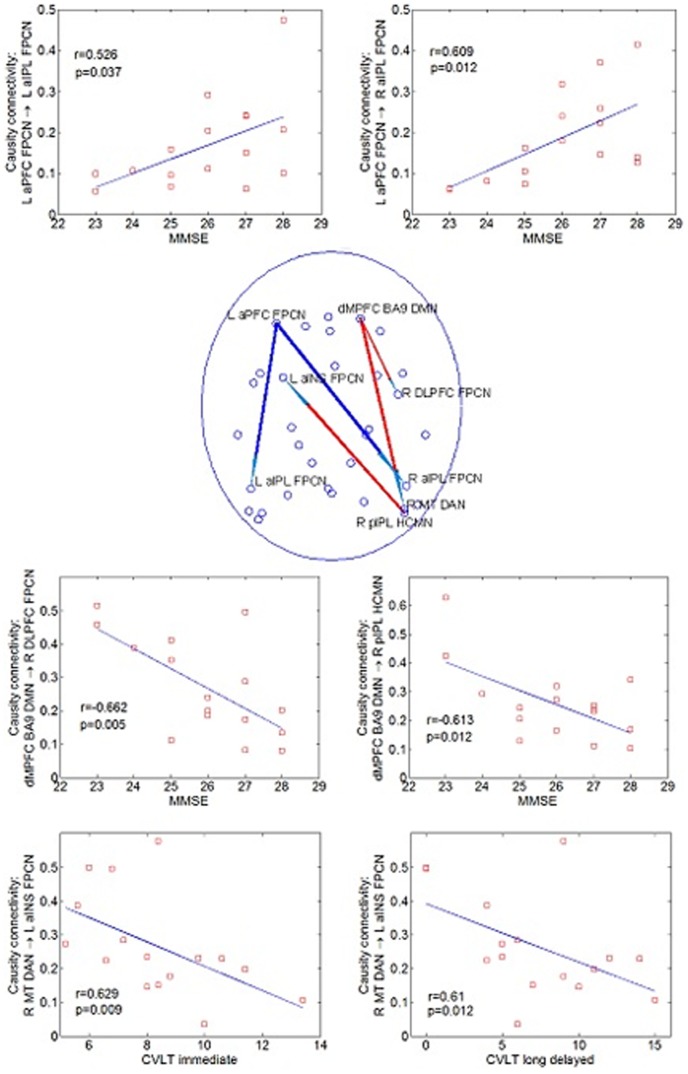
Pathways (Blue = NC>aMCI; Red = aMCI>NC) showed significant correlation between causal connectivity and neuropsychological measures (including MMSE and CVLT) in aMCI group, and scatter plots of these associations.

As shown in Figure 4, correlation analysis revealed that some causal paths showed significant correlation between CPGC values and neuropsychological performances in aMCI patients. The connectivity of two causal paths within FPCN, which were significantly decreased in aMCI as compared to NC, i.e., L aPFC FPCN→L aIPL FPCN (r = 0.526, *P* = 0.037) and L aPFC FPCN→R aIPL FPCN (r = 0.609, *P* = 0.012), were positively correlated with MMSE. While, the connectivity of the other three between-networks causal paths, which were significantly increased in aMCI as compared to NC, were negatively correlated with MMSE and CVLT measures. The causal connectivity of dMPFC BA9 DMN→R DLPFC FPCN was correlated with MMSE (r = −0.662, p = 0.005), dMPFC BA9 DMN→R pIPL HCMN correlated with MMSE (r = −0.613, p = 0.012), and R MT DAN→L aINS FPCN simultaneously correlated with CVLT (immediate) (r = −0.629, p = 0.009) and CVLT (long delayed) (r = −0.61, p = 0.012). All the other correlations were not significant.

### Causal Connectivity: Removing the Global Signal

An important issue in resting state fMRI data preprocessing is the removal of the global signal averaged from the whole brain. Although the nature of the global signal is unclear so far, it is widely admitted that the often discarded global component of fMRI fluctuations measured during the resting state is tightly coupled with underlying neural activity and might have biological origins [57]–[58]. There are some studies focusing on the influence of the global signal regression to the results of resting state functional connectivity, but the results are inconclusive. For example, some previous studies suggested that anti-correlations are artificially introduced into the data by the global signal removal [59]–[60], while some others suggested otherwise [61]. Further, the validity of global signal removal in effective connectivity analysis has not been studied.

Based on these considerations, we have reported the causal connectivity results based on data without the global signal regression. However, we also performed the causal connectivity analysis based on the data with global signal regression. It was found that, when the global signal was regressed out, the within-group results were significantly altered while the between-group difference patterns did not show much difference. Only the statistical values of some between-group difference paths were slightly changed.

### Causal Connectivity: Effect of Head Motion

The influence of head motion on resting state connectivity metrics, despite standard pre-processing steps to account for it, has been a matter of intense debate recently [62]. In order to address this issue, we obtained the mean value of frame wise displacement (FD) of the head for each subject. Using this as a quality control (QC) metric, we investigated whether they correlated with any of the causal connectivity paths in both groups. The motion of the head at each time instant was converted into a scalar quantity using the formula, FD_i_ = |Δd_ix_|+|Δd_iy_|+|Δd_iz_|+|Δα_i_|+|Δβ_i_|+|Δγ_i_|, where Δd_ip_ = d_(i−1)p_−d_ip_ where p is any of the 3 translational parameters (x,y,z) or rotational parameters (α, β, γ). The rotational displacements were converted from degrees to millimeters by estimating the displacement on the surface of a sphere of radius 50 mm. This made the assumption that the approximate mean distance from the center of the head to the cerebral cortex is 50 mm. Many recent studies have recommended the entire procedure described above involving the calculation of FD and subsequent correlation of its mean with connectivity metrics for either confirming or ruling out the influence of head motion on connectivity measures [62]–[66]. Mean FD was not significantly correlated (p>0.05) with any of the causal connectivity paths in either groups, demonstrating that head motion is unlikely to be a confounding factor in our analyses.

## Discussion

Many efforts have been made to detect early changes in aMCI before the onset of clinical dementia. The novelty of the present study lies in the fact that we have used directional connectivity methods to explore the underlying biological markers of MCI. The major findings of our study can be summarized as follows. First, aMCI was associated with opposing causal connectivity effects of decreased connectivity in the FPCN and HF-centered between-networks connections and simultaneous connectivity enhancements in frontal networks, DMN and some between-networks connections. Specifically, the causal connectivity alterations in aMCI patients were independent of GM atrophy in the present study. This may imply an extensive alteration in aMCI in information exchange and transfer between brain regions of different functional RSNs. Second, global signal regression only has a slight effect on the connectivity group difference. Third, cognitive impairment correlated with reduced FPCN causal connectivity, while cognitive compensation correlated with some between-networks causal connectivity in aMCI patients (see Figure 4). Together, these results may suggest that causal disconnection mechanisms result in a “genuine” functional perturbation over and above neuronal/synaptic loss in this disease. In the following sections, we discuss these results in comparison with previous literature and their functional roles in the cognitive impairment of aMCI patients.

### Anterior-Posterior Causal Connectivity: Decreased in FPCN

The decreased anterior-posterior causal connectivity, especially within the FPCN, in aMCI patients compared with healthy controls is in agreement with converging evidence from various modalities showing that such a network is affected preferentially by processes that are known to be associated with this disease [67]–[68], including resting state fMRI [20], task-fMRI [29], [40], and structural MRI [69]. Specifically, aPFC is at the apex of the control hierarchy [33] and has been implicated in integrating information from multiple sources [33], [51], [70], processing of internal states [71], and acting as a cognitive buffer [72]. Thus, the impairment of the path from aPFC to aIPL in aMCI may imply that the control information, if it is generated correctly, may not be intact when transferred to the posterior parietal cortex for maintenance and representation. This may be underlying the cognitive decline of aMCI patients, as evidenced by the fact that the effective connectivity of aPFC→aIPL is significantly correlated with MMSE.

### Anterior-Posterior Causal Connectivity: Increased in DMN

In the current study, the increased long-distance (anterior-posterior) causal links within DMN were observed. These results are consistent with previous resting state studies reporting increased PCC functional connectivity with frontal cortex (including medial frontal gyrus) [11], [16] in aMCI patients. These findings also seem to be logical given the failure to deactivate the DMN in aMCI patients during goal-directed processing [17], [73]–[74]. We thus postulated that the increased anterior-posterior causal connectivity in DMN may compensate decreased anterior-posterior causal connectivity in FPCN in aMCI patients.

Additionally, dMPFC has been implicated in integrating the external environment with stored internal representations [75]. Given the anatomical proximity of dMPFC to aPFC, the increased causal connectivity associated with dMPFC may compensate for the reduced connectivity associated with aPFC and hence offset some of the decreased abilities in executive functions in aMCI patients. This is further confirmed by the fact that the connectivity of dMPFC BA9 DMN→R DLPFC FPCN and dMPFC BA9 DMN→R pIPL HCMN are significantly correlated with MMSE scores of aMCI patients.

### HF Causal Disconnection

The impaired HF causal connectivity in aMCI further demonstrate the previous findings of HF functional disconnection in aMCI [10], [12], [19] and adds insights of aberrant information flow. The current results are also congruent with the structural atrophy (see two recent meta-analysis reviews: [76]–[77]) and regional glucose metabolism reductions [42]–[43] of HF in MCI. Specifically, we found most of regions showed decreased causal connectivity with right HF located in DMN, including PCC, bilateral pIPL and right DLPFC, which subserve memory functions together with HF [36], [75], [78]. Experiments of cognitively intact older individuals have also demonstrated that intrinsic functional connectivity between the hippocampus and PCC/PCu during the resting state is significantly correlated with performance of episodic memory tasks [79]. Thus, consistent with previous reports, the disrupted HF effective connectivity may contribute to memory impairment of aMCI patient.

Additionally, we also observed an increased connectivity within HCMN, i.e., L HF HCMN→L pIPL HCMN. Note that this increase was observed in the left hemisphere while large-scale decreases were associated with R HF. This is an effect similar to increased HF functional connectivity [10], [19] and HF hyper-activation [17], [80]–[82] identified in previous studies, and may suggest a compensatory mechanism to decreased causal connectivity associated with R HF and consequent memory loss in aMCI patients.

### Enhanced Within-Frontal Causal Connectivity

We observed increased short-distance causal connections within the right frontal cortex in the aMCI group. Previous studies have reported increased activity in the right DLPFC [39], [83], and increased functional connectivity between the right prefrontal cortex and the left frontal areas and other regions [11], [20], [41] in MCI patients using resting state or various cognitive tasks (e.g., inductive reasoning and memory encoding). The increased right frontal connectivity in aMCI patients relative to controls suggests that patients with aMCI may rely on additional neural resources in the right prefrontal regions to compensate for reduced executive function in the advanced phase of the disease. This hypothesis is reinforced by our observation that more severe cognitive deficits (as measured by MMSE) are associated with increased within-frontal connectivity (i.e., dMPFC BA9 DMN→R DLPFC FPCN) in aMCI patients.

### Between-Networks Causal Connectivity

Most of previous studies only focus on a single functional network [10]–[11], [16], [19]–[20] or several networks separately [21], [84] in aMCI patients. A recent resting state study of aMCI patients using graph analysis [44] identified the connections targeted by the disease in the inter-module (network) links among different functional systems. In the present study, we found both decreased and increased between-networks causal connections. Further, some altered inter-network causal connectivity (e.g., DMN→FPCN, DMN→HCMN, DAN→FPCN) were correlated with cognitive behavioral measures of aMCI patients (see Figure 4). Given that the DMN contributes to episodic memory [15], [78], the increased DMN→HCMN causal connectivity may reflect compensation for episodic memory impairment in aMCI patients. The increased DAN→FPCN causal connectivity was significantly correlated with episodic memory measurement in aMCI, which may suggest that patients tend to recruit more attention and executive control resources to support episodic memory processes.

The most intriguing finding is the causal connectivity between DMN and FPCN, which is actually anti-correlated with each other in healthy controls, but positively correlated in patients with aMCI, and negatively associated with the general cognitive decline of patients. Recent studies reported that the integrity of FPCN (specifically, dACC and aINS) is necessary for the efficient regulation of activity in the DMN [85]–[86] but in the opposite direction DMN may send signals that interfere with task control and leading to degraded behavioral performance [86]. Although using different data modality (DTI, task fMRI, respectively) from this study (i.e., resting sate fMRI), these studies have uncovered the causal interactions between FPCN and DMN. Thus, the increased synchronization between the two networks in aMCI patients may imply a mechanism to compensate for the abnormal within-network activity (as aforementioned, decreased causal connectivity within FPCN and increased causal connectivity within DMN in patients) and thus be helpful for maintaining the cognitive performance.

Together, the increased inter-network causal connectivity seen in these patients may have a role in maintaining cognitive efficiency in the presence of cognitive decline and memory impairment. The presented results extend previous findings and further demonstrate the inter-network directional connectivity in aMCI patients.

### Limitations

There are still technical and biological limitations in the current study. The first limitation is the relatively small sample size of patients. Future larger studies in aMCI patients are still required to validate the present findings, as larger samples tend to minimize the probability of errors, maximize the accuracy of population estimates, increase the generalizability of the results and thus increase the power to study effects of interest. The second limitation is the head motion issue. Although the head motions were regressed out during the data preprocessing, they were not significantly different between the two groups (P>0.05), and mean frame-wise displacement of the heads of individual subjects [62]–[66] were not significantly correlated with causal connectivity metrics in either groups, we cannot unequivocally exclude the possibilities of the confound effect of the head motion on the final result. Thus, we will hope to replicate these results in other datasets in the future. The third limitation is that aMCI patients may have different progressive trajectories, with some ultimately developing AD and others who do not. A longitudinal approach, which would allow those aMCI patients really prodromal to AD to be identified, would be required to directly test whether the current findings only hold for a subset of aMCI or apply to all kinds of aMCI patients. In the future, we will ask subjects to report their spontaneous thoughts after scanning such as using resting state questionnaire [87], which may contribute to exploring the behavioral group differences in such a resting state study. Finally, recent studies have demonstrated that there is clinically meaningful information in frequency bands greater than 0.08 Hz up to 0.3 Hz [88]. Such studies typically use a sub-second TR (645 ms in the case of Liao et al [88]) such that confounds arising from aliasing of high frequency physiological fluctuations into lower frequency bands can be minimized. However, given the fact that we used a TR of 2 s, we were unable to investigate frequencies beyond the conventionally used range of 0.01 to 0.08 Hz because we cannot be sure that we will be able to effectively account for aliased, in-band physiological noise. However, future studies should acquire data with sub-second TRs in an effort to include the 0.08 to 0.3 Hz range in their analyses.

Despite these limitations, this study showed that aMCI is associated with causal connectivity alterations of large-scale functional brain networks which is independent of gray matter atrophy, and extends our knowledge well beyond the previous FC-related findings. The current findings provide novel insights into the pathophysiological mechanism of aMCI and highlight the potential of using both intra- and inter-network causal connectivity as imaging biomarkers.
